# CaMKII comes of age in cardiac health and disease

**DOI:** 10.3389/fphar.2014.00154

**Published:** 2014-07-03

**Authors:** Eleonora Grandi, Andrew G. Edwards, Anthony W. Herren, Donald M. Bers

**Affiliations:** ^1^Department of Pharmacology, University of California, DavisDavis, CA, USA; ^2^Institute for Experimental Medicine, Oslo University Hospital UllevålOslo, Norway; ^3^Simula Research LaboratoryLysaker, Norway

**Keywords:** heart failure, arrhythmia, ion channels, phosphorylation, hypertrophy

## Translational perspective

Almost four decades since its initial discovery in brain (Schulman and Greengard, 1978), the multifunctional Ca^2+^ and calmodulin-dependent protein kinase II (CaMKII) has now emerged as a key signaling molecule in the heart. The isoform that predominates in heart, CaMKIIδ, directly regulates expression and function of several of the main cardiac ion channels and Ca^2+^ handling proteins (Bers and Grandi, 2009). CaMKII-dependent effects are thought to orchestrate many of the electrophysiologic and contractile adaptations to common cardiac stressors, such as rapid pacing, adrenergic stimulation, and oxidative challenge. In the context of disease, CaMKII has been shown to contribute to a remarkably wide variety of cardiac pathologies, including myocardial hypertrophy, ischemia, heart failure (HF), and arrhythmia. CaMKII expression is increased in patients with HF (Hoch et al., 1999), and elevated CaMKII expression and activity have been implicated in the transition to HF (Zhang et al., 2003). Indeed, inhibiting CaMKII appears to reduce arrhythmias and pathological signaling, which makes this kinase a promising new therapeutic target (Anderson et al., 2011).

## Overview of the research topic

This series reviews the molecular physiology of CaMKII and discusses the impact of CaMKII on heart function. The subjects were chosen to summarize the current state of our knowledge of various aspects of CaMKII-mediated control of myocyte physiology, with in-depth focused reviews by recognized leaders in these areas.

To start off, a group of articles examines the biochemistry of CaMKII activation and the pharmacology of its inhibitors. Recent efforts to characterize the individual pathways of CaMKII activation in the heart are reviewed. These studies improved our understanding of the downstream pathological consequences of these specific pathways (Erickson, 2014). Indeed, the promise of CaMKII inhibition for simultaneous prevention of HF progression and development of arrhythmias justifies the development of more specific CaMKII inhibitors that will further both basic research studies and drug development (Westenbrink et al., 2013). Thus, the kinase structure and possible sites for its inhibition (Pellicena and Schulman, 2014) are illustrated. It is also becoming clear that design of therapeutic intervention requires an improved understanding of the specific roles and function of the CaMKIIδ subtypes, δ B and δ C, as discussed by Gray and Heller Brown (2014).

A second group of articles reviews the large body of work describing CaMKII-specific interaction with its numerous intracellular targets, many of which play important roles in modulating cardiac excitation contraction coupling (ECC). CaMKII involvement at multiple levels in ECC indicates that it is an important modulator of both electrophysiological and contractile properties in the heart. Active CaMKII phosphorylates several Ca^2+^ handling proteins including ryanodine receptors (RyR2) (Camors and Valdivia, 2014), phospholamban (Mattiazzi and Kranias, 2014), and L-type Ca^2+^ channels (Bers and Morotti, 2014). In addition, non-Ca^2+^ transporters such as sarcolemmal Na^+^ (Grandi and Herren, 2014) and K^+^ (Mustroph et al., 2014) channels are regulated by CaMKII. This in turn influences myocyte Ca^2+^ regulation and also confers further Ca^2+^-dependence to a variety of electrophysiological processes. In combination these effects create a complex and non-linear feedback system that necessitates quantitative computational approaches. As such, multiscale models have played an important role in advancing our understanding of CaMKII function in cardiac ECC, particularly by being able to both reconstruct the details of local signaling events within the cardiac dyad, and predict their functional consequences at the level of the whole cell (Greenstein et al., 2014). CaMKII also regulates Ca^2+^ handling proteins that are thought to contribute relatively little to ECC, the best example of which is the inositol 1,4,5-trisphosphate receptor (IP3R), as reviewed by Camors and Valdivia (2014). This regulatory role is thought to be key in modulating IP3R-mediated Ca^2+^ release in the regulation of cytosolic and nuclear Ca^2+^ signals (Hohendanner et al., 2014). These mechanisms are suspected to be involved in coupling cytosolic and SR Ca^2+^ handling to adaptive transcriptional and epigenetic processes. Indeed, in addition to the above-mentioned acute effects due to phosphorylation of cytosolic proteins, CaMKII has been shown to influence chronic physiological and pathological processes, particularly those contributing to the ventricular remodeling that leads to HF (Kreusser and Backs, 2014).

Finally, the integrative role of this enzyme in cardiac physiology and disease is discussed. As described above, the multifunctional nature of CaMKII causes this integration to be very challenging, and the development of detailed multiscale computational models has greatly improved our ability to describe how molecular actions can impact tissue and organ function. The review by Onal et al. (2014) dissects how these systems biology approaches have contributed to linking CaMKII activity in single myocytes to observable tissue-level arrhythmogenic outcomes in cardiac disease. Experimental disease models that incorporate CaMKII overexpression clearly demonstrate a link between its excessive activity and arrhythmias associated with congenital and acquired heart disease. Vincent et al. (2014) review CaMKII involvement in both types of disease, and argue that the importance of CaMKII phosphorylation at RyR2 across a variety of disease models suggests that this molecular interaction may be a point of mechanistic convergence in many forms of cardiac arrhythmia. In addition to its abundantly studied ventricular consequences, such as in HF and ischemia/reperfusion challenge (Bell et al., 2014), CaMKII has emerged as a key determinant of sinoatrial node dysfunction (as well as a central regulator of physiological sinoatrial node responses) (Wu and Anderson, 2014; Yaniv and Maltsev, 2014). Recently, a significant effort has also been invested in describing the role played by CaMKII in atrial fibrillation, where the many actions of CaMKII are superimposed upon the unique electrophysiologic and structural characteristics of the diseased atrium (Heijman et al., 2014).

## Conclusions and future directions

CaMKII is an abundant signaling molecule in myocardium, where it integrates and transduces cellular Ca^2+^ signals into physiological responses in heart. Under various pathological stresses, CaMKII hyperactivity appears to be an important component of disease signaling. However, in the context of complex cardiac disease, such as HF, multiple signaling pathways are likely to be altered, many of which might crosstalk with CaMKII signaling. Thus, the interplay of various mechanisms of CaMKII activation, particularly during the development of cardiovascular diseases, is an important open avenue of investigation. As a complicating factor, CaMKII itself contributes to the regulation of intracellular signaling processes, such as mitochondrial function (Joiner and Koval, 2014), thus forming potential feedback loops leading to further CaMKII activation.

Recent findings that CaMKII inhibition ameliorates HF and suppresses arrhythmias suggest that developing specific CaMKII inhibitory drugs may be a new therapeutic approach to these diseases. However, current pharmacological tools are limited. Development of new inhibitors (Pellicena and Schulman, 2014) will enable preclinical proof-of-concept tests and clinical development of successful lead compounds, as well as improved research tools to more accurately examine and extend knowledge of the role of CaMKII in cardiac health and disease.

### Conflict of interest statement

The authors declare that the research was conducted in the absence of any commercial or financial relationships that could be construed as a potential conflict of interest.
